# Traumatic Hallux Varus Treated by Minimally Invasive Extensor Hallucis Brevis Tenodesis

**DOI:** 10.1155/2015/179642

**Published:** 2015-12-17

**Authors:** C. N. Cheung, T. H. Lui

**Affiliations:** Department of Orthopaedics and Traumatology, North District Hospital, 9 Po Kin Road, Sheung Shui, New Territories, Hong Kong

## Abstract

A case of traumatic hallux varus due to avulsion fracture of the lateral side of the base of proximal phalanx was reported. The lateral instability of the first metatarsophalangeal joint was believed to be due to the disruption of adductor hallucis function. It was successfully managed by minimally invasive extensor hallucis brevis tenodesis.

## 1. Introduction

Hallux varus has been described as a triplane deformity consisting of a medially deviated, hammered hallux in a varus rotation [1–3]. It is often associated with pain, functional impairment, difficulty with shoe gear, and cosmetic dissatisfaction. This deformity of the first metatarsophalangeal joint (MTPJ) is either congenital or acquired. It is usually iatrogenically induced and is most commonly associated with hallux valgus surgery [3]. Traumatic hallux varus was uncommonly reported [2–5]. We reported a case of traumatic hallux varus that was successfully treated with minimally invasive extensor hallucis brevis (EHB) tenodesis [6].

## 2. Case Presentation

**Figure 1:**
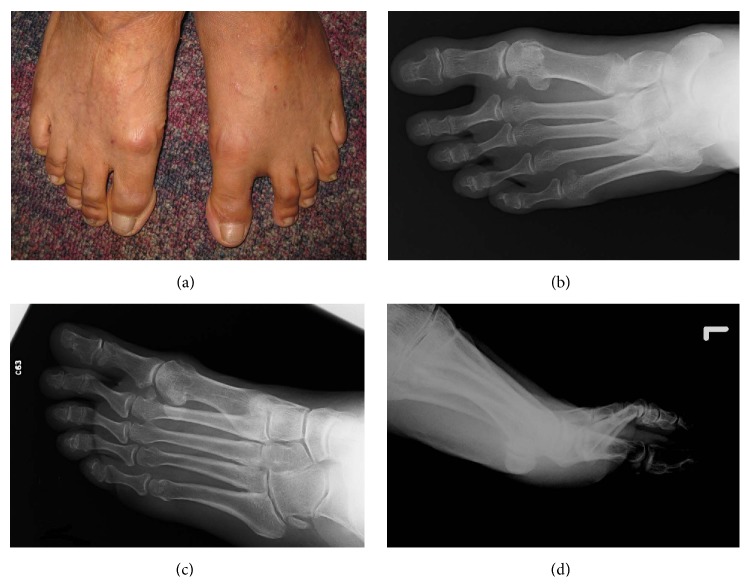
Clinical photos of this patient showed left hallux varus deformity (a). Radiographs showed avulsion fracture at the lateral side of base of proximal phalanx (b, c, and d).

A 68-year-old lady had her left great toe hit by a door over the lateral side of the toe. She noticed bruise and pain over her left great toe afterwards. She consulted the family doctor and was treated as soft tissue contusion with buddy splint and analgesics. However, she still experienced severe great toe pain with shoe gear and walking. Radiograph of her left foot was then taken and showed avulsion fracture of the lateral side of the base of her left great toe. She was referred to our clinic 5 weeks after the injury. Clinical examination showed left hallux varus (Figure 1) and increased ease of passive abduction of the left great toe as compared to the right side (see Video 1 in Supplementary Material available online at http://dx.doi.org/10.1155/2015/179642). Operative reconstruction of the lateral stabilizer of the 1st MTPJ was suggested but she refused initially. However, her left great toe symptoms persisted and she finally agreed about operation. Operation was performed 5 months after the injury. First MTP arthroscopy showed intact cartilage with diffuse synovitis and arthroscopic synovectomy was performed. Minimally invasive EHB tenodesis was then performed to correct the hallux varus. Plantar plate tenodesis was also performed to correct the symptomatic clawing of the 2nd toe.

## 3. Technique

**Figure 2:**
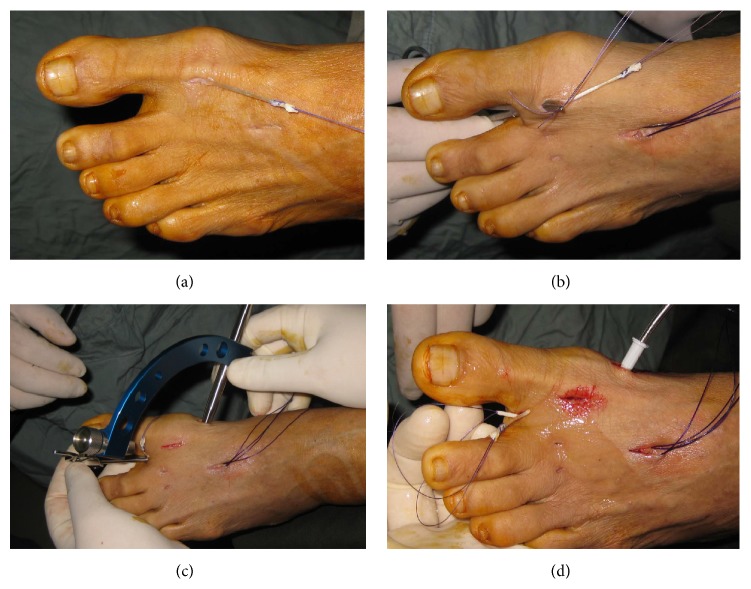
(a) The EHB tendon graft was retrieved to the dorsolateral portal wound and a stay stitch was applied. (b) The stay stitch and the tendon graft were retrieved to the toe web wound with a hemostat. Note that plantar plate tenodesis was also performed for the claw second toe. (c) A bone tunnel was made by means of the Micro Vector drill guide. (d) The stay stitch was put into the angiocath cannula and suction was applied at the other end. The stitch and the tendon graft can then be brought under the intermetatarsal ligament through the bone tunnel to the medial side of the neck of the first metatarsal.

The patient was put in supine position and a pneumatic thigh tourniquet was applied. A small incision was made at the dorsolateral side of the toe extensor tendons at the level of first metatarsophalangeal joint line which was the same as the dorsolateral portal of 1st MTP arthroscopy. The EHB tendon was identified at this wound. A proximal wound was made at the proximal end of the EHB tendon, and the tendon was cut and retrieved to the distal wound. A wound was made at the web space and the tendon graft was retrieved to the toe web wound. Another wound was made at the medial side of the metatarsal 1.5 cm proximal to the metatarsophalangeal joint. The wound was retracted distally to expose the medial capsule of the joint. The small capsulotomy was then made and the medial capsule was stripped from the bone with a small periosteal elevator. The whole medial capsule was stripped away from the bone from dorsal to plantar, including the proximal edge. A bone tunnel was then made through this wound with a 3.2 mm drill directing obliquely from the dorsomedial cortex to the plantar, distal, and lateral aspect of the first metatarsal. The direction of drilling was guided by a Micro Vector drill guide (Smith & Nephew) with the probe through the toe web wound just underneath the intermetatarsal ligament. A long Angiocath was passed through the bone tunnel and grasped by a hemostat through the toe web wound. The needle of the angiocath was removed and the plastic cannula of the angiocath was then brought to the toe web wound. The stay stitch was put into the cannula and suction was applied at the other end. The stitch and the tendon graft can then be brought under the intermetatarsal ligament through the bone tunnel to the medial side of the neck of the first metatarsal (Figure 2). The hallux varus deformity was then corrected by tensioning of the graft and the first metatarsal was transfixed with a 1.6 mm K wire. The graft was sutured to the abductor hallucis under tension.

**Figure 3:**
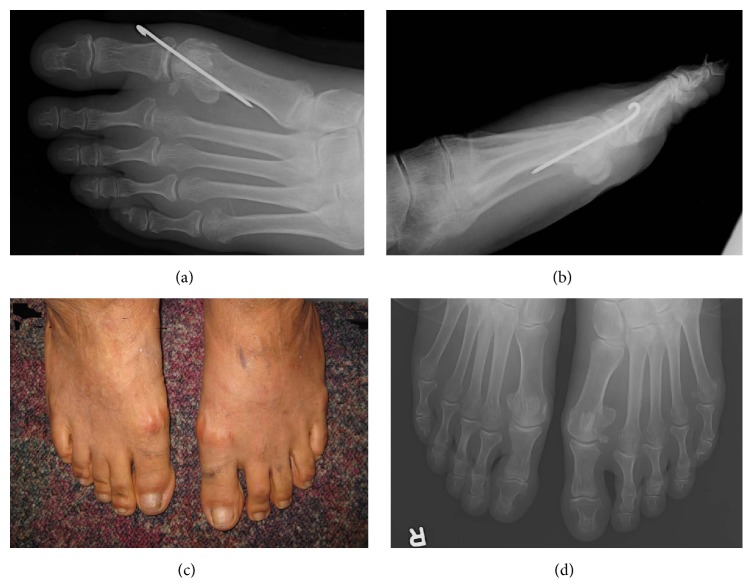
(a, b) Postoperative X-rays showed the correction of the hallux varus and the K wire in situ. (c) Clinical photos 31 months after the operation showed the correction maintained. (d) Radiograph showed the correction maintained and no degenerative change of the 1st MTPJ 31 months after the operation.

**Figure 4:**
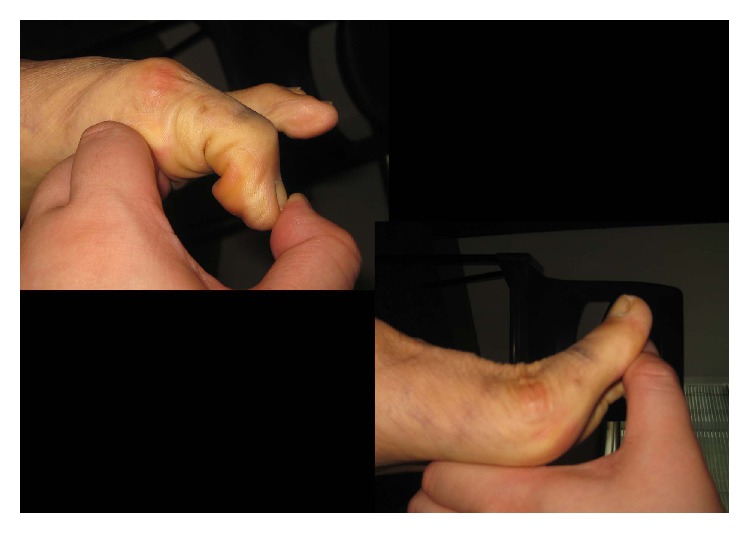
Postoperatively, the passive dorsiflexion and plantar flexion range of motion of the first MTPJ was satisfactory.

Postoperatively, the patient was advised on nonweight bearing walking. The K wire was removed and the patient was allowed weight bearing walking with wooden base sandal 4 weeks after the operation. She can resume normal shoe gear 2 months after the operation. There was no complication noted. Upon the latest follow-up 31 months after the operation, there was no more great toe pain and the hallux varus deformity was corrected (Figure 3). The lateral stability of the 1st MTPJ was restored (Video 2) and dorsiflexion-plantar flexion motion of the great toe was satisfactory (Figure 4).

## 4. Discussion

The function of the adductor hallucis was believed to be disrupted in this case through avulsion of its phalangeal insertion. One of the functions of the adductor hallucis muscle is to provide transverse plane stability of the hallux at the first MTPJ. To accomplish this function, the adductor hallucis acts to balance the pull of its antagonist muscle, abductor hallucis. Hallux varus can occur after traumatic rupture of the adductor hallucis, inducing muscle imbalance around the first metatarsophalangeal joint from an unopposed abductor hallucis muscle [2–5]. Besides development of hallux varus deformity, the loss of normal function of the adductor hallucis muscle would cause the loss of stability of the proximal phalanx against the metatarsal head and against the ground reactive forces during propulsion. The resulted abnormal first metatarsophalangeal joint motion may lead to early-onset osteoarthritis [3, 7]. Surgical reattachment of the adductor hallucis to the proximal phalanx through bone tunnel has been described [5]. However, it may be not feasible in case of delayed presentation as the tendon can be severely contracted and normal tendon length is unattainable [3]. Surgical plication of the lateral capsule was also described but the correction may not be strong enough and may need suture anchor to augment the repair [3]. Myerson [7–9] used an extensor hallucis brevis tenodesis procedure to restore the transverse plane stability. It is indicated in case of symptomatic hallux varus with flexible metatarsophalangeal and interphalangeal joints and absent arthritis of the first metatarsophalangeal joint [9]. Through a dorsal longitudinal wound, the tendon is detached proximally and passed underneath the intermetatarsal ligament and finally attached to the first metatarsal under tension through a bone tunnel. It acts as a static tenodesis to extension and varus forces on the hallux when the transverse ligament is used as a pulley [8]. The technique used in this case is a minimal approach of Myerson's technique. The same surgical principle was applied through small wounds in order to minimize the surgical trauma [6]. This minimally invasive procedure has been used for correction of hallux varus deformity following hallux valgus surgery. This is the first report of utilization of this procedure to treat posttraumatic hallux varus. This is a tenodesis procedure, that is, nonanatomically reconstruction of the lateral constraint of the first metatarsophalangeal joint. The motion of the joint was expected to be diminished. This unexpectedly good motion of the first metatarsophalangeal joint in this case may be because of pinning of the joint before tensioning of the graft. This can avoid overconstraining of the joint and development of iatrogenic hallux valgus or joint motion restriction. However, pinning of the joint can cause iatrogenic damage to the articular cartilage of the first metatarsophalangeal joint and risk of pin breakage before its removal. Another approach would be appropriate postoperative bandaging to maintain the hallux position for 4–6 weeks. This case report illustrated the feasibility of minimally invasive approach of EHB transfer to correct posttraumatic hallux varus deformity. Larger series with longer follow-up is needed to confirm the benefits of this minimally invasive approach.

## Supplementary Material

Video 1: There was increased ease of passive abduction of the left great toe as compared to the right side which signified laxity of the lateral collateral ligament of the first metatarsophalangeal joint.
Video 2: the stability of the first metatarsophalangeal joint to varus stress was restored after extensor hallucis brevis tenodesis.
